# Role of Progesterone Receptor Level in Predicting Axillary Lymph Node Metastasis in Clinical T1-T2N0 Luminal Type Breast Cancer

**DOI:** 10.3390/medicina61040710

**Published:** 2025-04-12

**Authors:** Mihriban Erdogan, Canan Kelten Talu, Zeliha Guzeloz, Gonul Demir, Ferhat Eyiler, Seval Akay, Ezgi Yilmaz, Olcun Umit Unal

**Affiliations:** 1 Department of Radiation Oncology, University of Health Sciences Izmir College of Medicine and Izmir City Hospital, 35540 Izmir, Turkey; zelihaguzeloz@yahoo.com (Z.G.); gonuldemir1990@gmail.com (G.D.); ferhateyiler@gmail.com (F.E.); 2 Department of Pathology, University of Health Sciences İzmir Tepecik Training and Research Hospital, 35020 Izmir, Turkey; ecanankelten@gmail.com (C.K.T.); ezgiyilmaz.tp@gmail.com (E.Y.); 3 Department of Medical Oncology, Izmir City Hospital, 35540 Izmir, Turkey; drsevalsekerler@hotmail.com (S.A.); drolcun@hotmail.com (O.U.U.)

**Keywords:** breast cancer, estrogen receptor, progesterone receptor, axillary lymph node, metastasis

## Abstract

*Background and Objectives*: Axillary lymph node metastasis and the number of metastatic lymph nodes are important prognostic factors which are directly related to overall survival in women with breast cancer. Several factors have been identified to predict the likelihood of axillary lymph node metastasis in early-stage breast cancer. High PR expression is often more prevalent in the luminal A subgroup, which is associated with a better prognosis. The aim of this study was to determine the relationship between the percentage of PR expression and the likelihood of axillary metastasis in Her-2-negative, clinical T1-T2N0 luminal type breast cancer. *Materials and Methods*: A hundred and ninety-nine cases with luminal type, Her-2-negative, clinically and radiologically axilla-negative T1-T2 breast cancer who received radiotherapy were evaluated retrospectively. The pathological specimens were assessed by an experienced pathologist. *Results:* The statistical evaluation showed that tumor diameter greater than 2 cm, (*p* = 0.003), presence of lymphovascular invasion (*p* = 0.001), and PR expression level below 80% (*p* = 0.037) were identified as significant predictors of lymph node positivity in breast cancer patients. *Conclusions:* Percentage of progesterone receptor expression along with other molecular biological markers and clinicopathological parameters should be evaluated altogether when predicting axillary metastasis risk before surgery.

## 1. Introduction

Breast cancer is one of the most common cancers in women, and its incidence is increasing every year. Axillary lymph node metastasis and the number of metastatic lymph nodes are important prognostic factors directly related to overall survival in women with breast cancer [1]. Sentinel lymph node biopsy (SLNB) is one of the minimally invasive techniques that can confirm the presence of metastasis in regional lymph nodes [2,3]. Currently, it is a standard procedure for clinically N0 breast cancer of T1-T2 stage [4]. Several clinicopathological factors have been identified to predict the likelihood of axillary lymph node metastasis in early-stage breast cancer. These include tumor size, presence of lymphovascular invasion, tumor grade, hormone receptor status, age, and molecular subtypes.

Lymphovascular invasion is observed in one-third of breast tumors and has been shown in studies to be a negative prognostic factor for survival and an independent predictive factor for nodal spread [5,6,7]. Additionally, similar studies have reported that as tumor size increases, the likelihood of lymphatic invasion increases, and the probability of axillary metastasis shows a positive correlation with tumor size [6,7,8].

The importance of Her-2 in cell proliferation and differentiation is now well recognized. Her-2 positivity has been found to be higher in estrogen receptor (ER)/progesterone receptor (PR)-negative, lymph node-positive, and highly proliferative cancers [9,10].

Ki-67, a proliferation marker, is a nuclear protein that has been studied in many cancer types, including breast cancer. In the St. Gallen consensus, Ki-67 has been used to distinguish luminal A from Her-2-negative luminal B in molecular subtype classification (Ki-67 < 14% and PR more than 20% vs. Ki-67 ≥ 14%) [11]. However, a clear relationship between Ki-67 expression and sentinel lymph node metastasis in breast cancer has not yet been established. Another factor reported with controversial and varied results is the presence of a high grade [6,7].

Estrogen and progesterone are steroid hormones that play a significant role in the formation and progression of breast cancer, with about 70% of breast cancers testing positive for them [12]. ER/PR-positive tumors respond better to hormone therapy and have a better prognosis. PR indicates the presence of the functional estrogen receptor pathway and suggests that endocrine therapy will be more successful [13]. Therefore, it has been observed that cancer-specific survival is better in ER/PR-positive cases compared to tumors that are ER-positive and PR-negative [14,15]. Moreover, high PR expression is often more prevalent in the luminal A subgroup, which is associated with a better prognosis [16].

A decrease in PR expression may indicate a transition from a favorable prognosis to a relatively poor prognosis, potentially increasing the risk of axillary metastasis. This has been an observation that caught our attention in clinical practice. While it is known that decreased progesterone receptor levels worsen prognosis, it may also be a factor that increases the risk of axillary lymph node metastasis. The specific threshold below which the progesterone receptor level increases this risk has not yet been determined. Once this level is identified, surgeons, in early-stage luminal type breast cancer where surgical decisions are made, may anticipate an elevated risk of axillary metastasis based on reduced progesterone receptor levels, along with other established risk factors.

In this study, we aimed to determine the relationship between percentage of progesterone receptor expression and the likelihood of axillary metastasis in Her-2-negative, clinical T1-T2N0 luminal type breast cancer.

## 2. Materials and Methods

### 2.1. Study Design and Ethics

This study is a retrospective, single-center study that included patients with early-stage breast cancer without clinically identifiable axillary lymphadenopathy. Clinically and radiologically T3, T4, N+, M+, triple (−), Her-2 (+), and ER (−),/PR (+) patients, as well as those who received neoadjuvant chemotherapy before surgery, were excluded from this study. All pathology specimens were re-evaluated by an experienced pathologist. To identify factors associated with lymph node metastasis, the following variables were evaluated: tumor size; lymphovascular invasion; histological type; perineural invasion; PR level; Ki-67 proliferation index; and immunohistochemical subgroup (ER+/PR+ vs. ER+/PR−). Since a predictive cut-off value for progesterone is not known, four groups were created based on progesterone levels, classified as below and above 10%, 20%, 50%, and 80%. This study was conducted in accordance with the Declaration of Helsinki and approved by the Institutional Review Board (IRB) Manager (protocol no: 2023/07-03).

### 2.2. Data Collection and Inclusion Criteria

**Figure 1:**
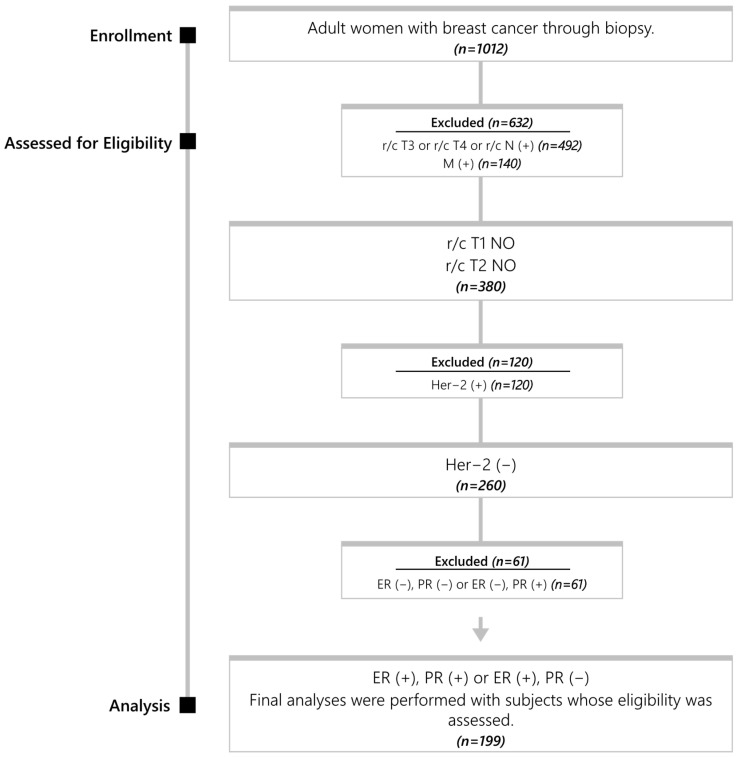
Flowchart. r/c: radiology/clinic; M: metastasis; ER: estrogen receptor; PR: progesterone receptor.

Patient records with breast cancer screened for the ICD 10 code C50.9 were reviewed between January 2019 and December 2023. Finally, 199 cases with luminal type, Her-2-negative, clinically and radiologically axilla-negative T1-T2 breast cancer who received radiotherapy were included (Figure 1).

### 2.3. Pathological Evaluation

The pathology specimens obtained by core needle biopsy, breast conserving surgery, and/or mastectomy were re-evaluated by an experienced pathologist. PR percentage levels were obtained from Tru-cut material and determined using the immunohistochemistry method.

According to the St. Gallen consensus, Ki-67 has been used to distinguish luminal A from Her-2-negative luminal B in molecular subtype classification (Ki-67 < 14% and PR more than 20% vs. Ki-67 ≥ 14%) [11].

### 2.4. Statistical Analysis

The statistical analyses were performed using IBM SPSS Statistics Software for Windows version 24 (IBM Corp, Armonk, NY, USA). *p* < 0.05 was considered statistically significant. The groups were compared using the Chi-square test and logistic regression analysis. The significance of differences in categorical variables was evaluated using the Chi-square test. Univariate and multivariate logistic regression analyses were conducted to identify significant predictors of lymph node positivity in breast cancer patients.

## 3. Results

A total of 199 breast cancer patients who met the eligibility criteria were retrospectively analyzed. The clinical and pathological characteristics of the patients are summarized in Table 1.

All patients were female. The median age of the patients was 56 years [range 35–86], and 95.5% of them were over 40 years old. The right-sided and left-sided breast cancer rates were nearly equal (48.2% and 51.8%, respectively). The majority of cases (93%) were unifocal tumors. Mastectomy was performed in only 16 (8%) patients. Invasive ductal carcinoma was the most common histological subtype (89.9%), and 69.9% of patients had Grade I-II tumors. The median tumor size was 20 mm (range 3–50 mm). T2 breast tumors were found in 98 patients (49.2%), and T1 tumors were found in 101 patients (50.8%). Lymphovascular invasion (LVI) was present in only 29.6% of cases. The majority of tumors were ER+ and PR+ (93%). The rates of luminal A, luminal B, and unknown types of luminal disease were 33%, 60.5%, and 6.5%, respectively.

Of the total 199 patients, 146 (73.4%) underwent only SLNB, while 53 (26.6%) underwent axillary dissection (37 as a complementary procedure with SLNB, and 16 due to non-detection of sentinel lymph node). Lymph node positivity was detected in 52 (28.4%) of the patients who underwent SLNB and in 26 (49%) of those who underwent axillary dissection. The median number of removed lymph nodes with SLNB was two (range 1–9), and the median number of positive lymph nodes was one (range 1–8). The median number of removed lymph nodes with axillary dissection was 10 (range 2–35), and the median number of positive lymph nodes was 2 (range 1–24). All patients received hypofractionated or conventional fractionation breast/chest wall ± peripheral lymphatic radiotherapy with a median dose of 50 Gy (40–50 Gy) and a boost dose of 10 Gy.

**Figure 2:**
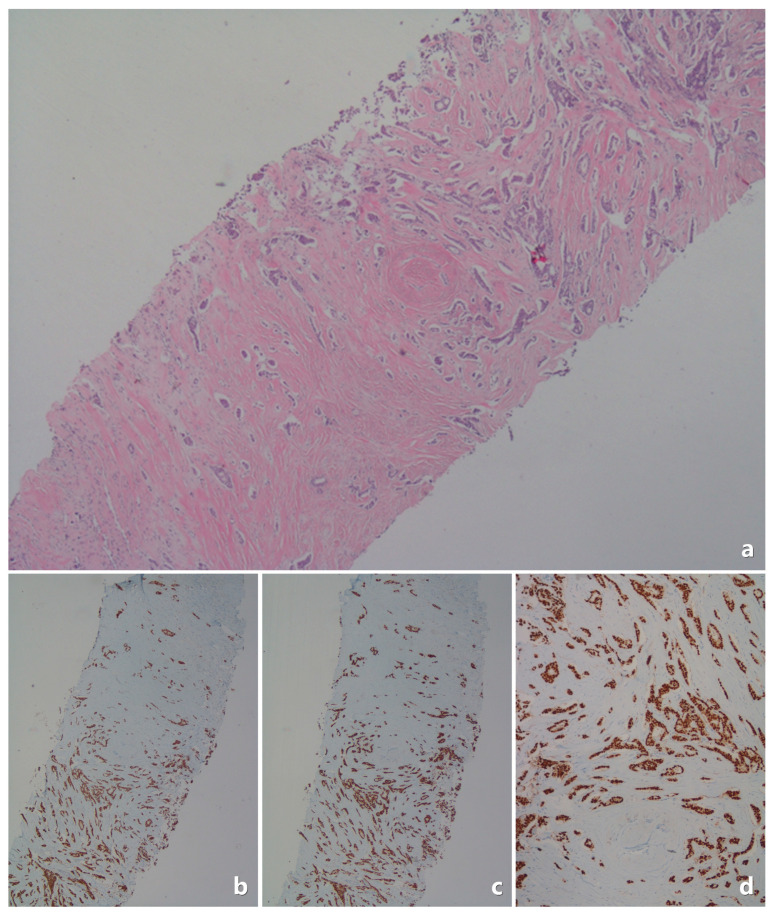
In the Tru-cut biopsy sample, the tumor is ER-positive, PR-positive (>80%), and the excision material shows negative lymph nodes. (**a**) Invasive breast carcinoma; core needle biopsy specimen. Tumor cells formed cord, trabeculae, and cribriform structures (H&E ×40). This case had no tumor metastasis in their sentinel lymph nodes (subsequent breast conservative surgery + sentinel lymph node biopsy). (**b**,**c**) Tumor cells showed diffuse and strong nuclear immunostaining for ERs and PRs; core needle biopsy specimen. (**b**) ER ×40; (**c**) PR ×40). (**d**) PR immunostaining; core needle biopsy specimen (PR ×100).

**Figure 3:**
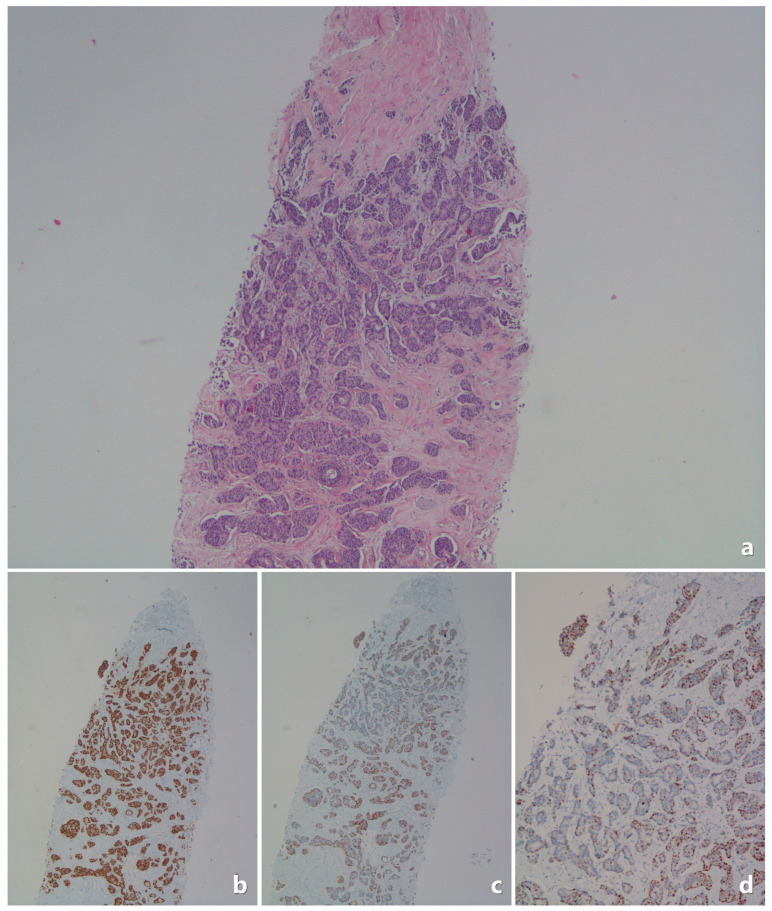
In the Tru-cut biopsy sample, the tumor is ER-positive and PR-positive (<80%). (**a**) Invasive breast carcinoma; core needle biopsy specimen. Tumor cells formed small solid islands and glandular structures (H&E ×40). This case showed tumor metastasis in their subsequent sentinel lymph nodes (is shown in Figure 4). (**b**) Tumor cells showed diffuse staining for ER; core needle biopsy (ER ×40). (**c**,**d**) Almost half of the tumor cells showed positivity for PR immunostaining; core needle biopsy ((**c**): PR ×40 and (**d**): PR ×100).

**Figure 4:**
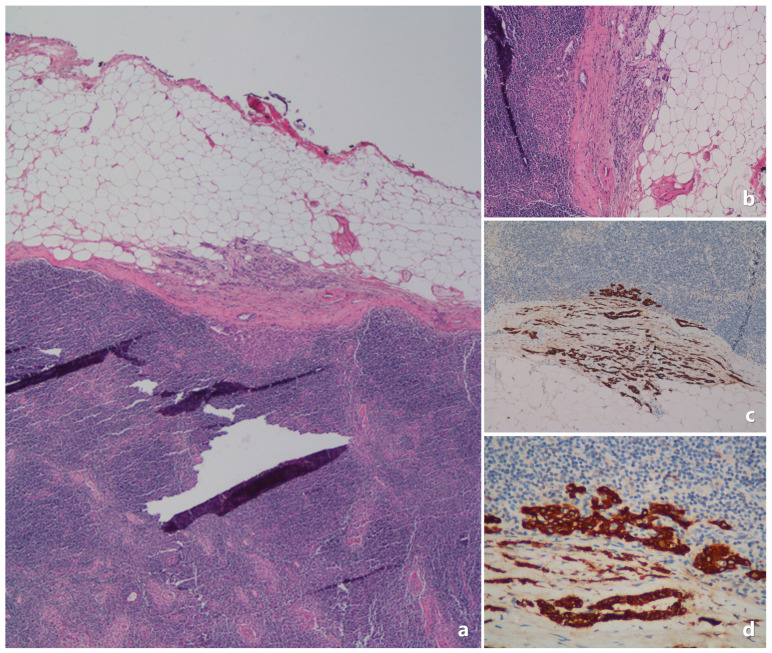
In the Tru-cut biopsy sample (Figure 3), the tumor is ER-positive, PR-positive (<80%), and the subsequent excision material shows an example of lymph node metastasis. (**a**) Tumor metastasis in a focal area in the lymph node capsule (H&E ×40). (**b**) Tumor cells formed glandular structures and trabeculae (H&E ×100). (**c**,**d**) The extent of the metastatic tumor focus in the capsule was revealed by pancytokeratin immunostaining ((**c**): pancytokeratin ×100; (**d**): pancytokeratin ×400).

**Figure 5:**
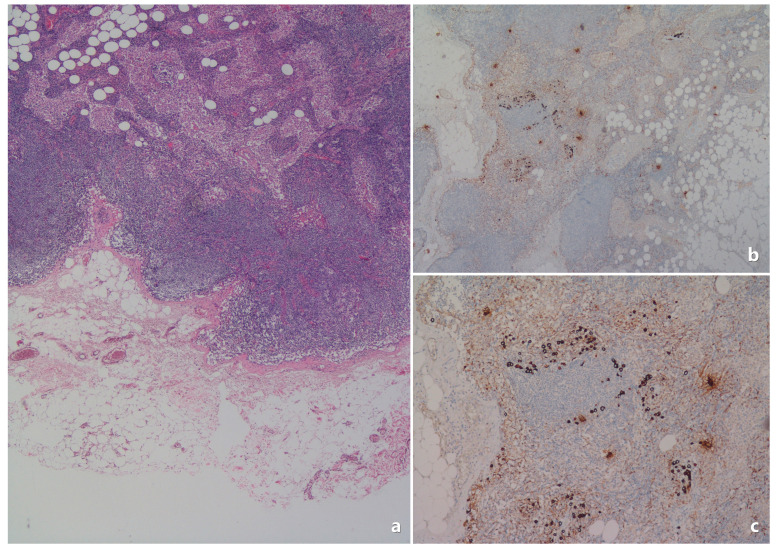
The lymph node metastases of invasive lobular carcinoma may not be very prominent and could be missed in H&E sections. (**a**) Axillary lymph node looks almost normal in appearance (H&E ×40). (**b**,**c**) Isolated tumor cells (invasive lobular carcinoma) were determined by pancytokeratin immunostaining ((**b**): pancytokeratin ×40; (**c**): pancytokeratin ×200).

The Tru-cut pathology specimens were re-evaluated by an experienced pathologist. Below are example images related to pathological evaluations (Figure 2, Figure 3, Figure 4 and Figure 5).

**Figure 6:**
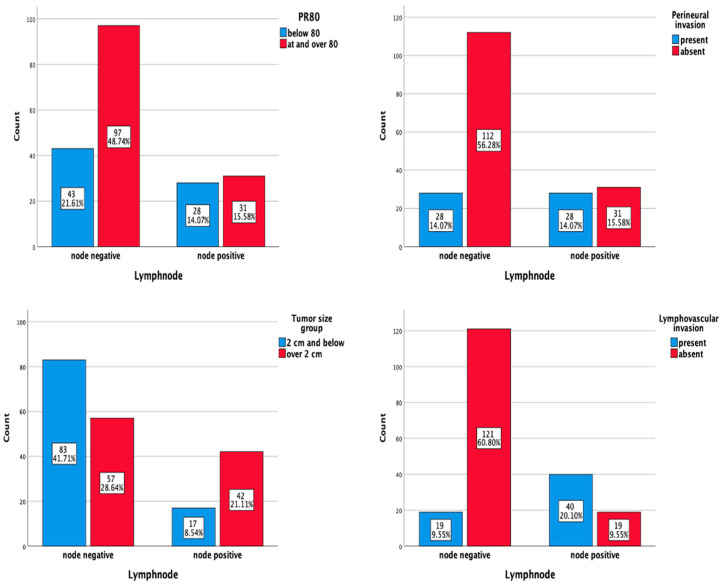
Significant factors according to the relationship between risk factors and lymph node status: tumor size, perineural invasion, lymphovascular invasion, and progesterone level <80%. N = 199 patients.

When risk factors that may be associated with higher lymph node metastasis risk were evaluated with Pearson’s Chi-square test, tumor size, lymphovascular invasion, PR value (<80% vs. ≥80%), and perineural invasion were found to be significant. Ki 67 index, immunohistochemical subgroup (ER+/PR+ vs. ER+/PR−), and other PR values (10%, 20%, 50%) were not statistically significant (Table 2, Figure 6). The rate of patients under 40 years old was 4.5%, and the rate of grade 3 patients was 5%. Therefore, evaluating these risk factors may not be appropriate.

According to the univariate logistic regression analysis, increasing tumor size (*p* < 0.001, OR: 3.59, 95% CI: 1.86–6.94), the presence of lymphovascular invasion (*p* < 0.001, OR: 13.33, 95% CI: 6.45–27.77), having progesterone levels below 80% (*p* = 0.026, OR: 2.03, 95% CI: 1.09–3.80), and the presence of perineural invasion (*p* < 0.001, OR: 3.61, 95% CI: 1.87–6.99) were found to be significant variables in predicting lymph node positivity (*p* < 0.05) (Table 3).

According to multivariate logistic regression analysis, a tumor diameter greater than 2 cm (*p* = 0.003, OR: 3.23, 95% CI: 1.47–7.04), the presence of lymphovascular invasion (LVI) (*p* < 0.001, OR: 1.11, 95% CI: 2.43–5.05), and a PR expression level below 80% (*p* = 0.037, OR: 2.30, 95% CI: 1.05–5.07) were identified as significant predictors of lymph node positivity in breast cancer patients; however, perineural invasion (PNI) was not found to be a significant predictor (Table 4).

## 4. Discussion

Axillary lymph node metastasis in breast cancer is a critical prognostic factor that significantly impacts survival, and it also guides decision-making for systemic treatments [17]. Because it influences both prognosis and treatment, this research focuses on the prediction of axillary lymph node metastasis.

Numerous factors have been studied to predict the risk of axillary metastasis in early-stage breast cancer. Among these, tumor size, lymphovascular invasion, and age have been identified as significant risk factors [18]. Therefore, physicians should be vigilant about axillary metastasis when there are factors such as increased tumor size, the presence of lymphovascular invasion, and younger age. Additionally, molecular subtypes such as Her-2-positive and triple-negative breast cancers are associated with increased lymphovascular invasion and higher rates of lymph node metastasis [19].

Recently, artificial intelligence studies applying deep learning methods to predict which preoperative clinicopathologic data may be associated with axillary lymph node metastasis in clinically N0 patients have attracted attention [20,21]. Zhang D. et al. investigated five different deep learning models that can predict macrometastasis in sentinel lymph nodes based on multiple clinicopathologic features in a population-based patient series of 18,185 patients with cT1-2 N0. The clinicopathologic data considered in this study were patient age, menstrual status, method of detection of the tumor mass, tumor size, number of invasive tumor foci, histological type, histological grade, ER, PR, CerbB2, and Ki-67 proliferation status, molecular phenotype of the tumor, and mean number of sentinel lymph nodes removed. Accordingly, tumor size alone was found to be the most important factor in predicting macrometastases in all deep learning models, while the number of invasive tumor foci was the second most important factor in the three deep learning methods. All of the deep learning models applied (especially the Transformer model) were reported to be superior to the logistic regression analysis in detecting macrometastases, provided that the sensitivity was not below 90% [20]. In another study in which the data of 2890 breast cancer patients were evaluated, when the TabNet model was applied, patient age, family history, tumor size, and core needle biopsy pathology findings and lymphovascular invasion detection were found to be among the characteristics associated with sentinel lymph node metastasis, while a significant association was reported between logistic regression analysis and unilateral/bilateral involvement of the tumor, age at first pregnancy, and progesterone values [21]. Some characteristics such as patient age, tumor location, size, grade, and ER/PR/Cerb-B2 status are among the parameters found to be significant in nomograms prepared to detect axillary lymph node metastasis in early-stage breast cancer [22,23,24].

In our study, we investigated which of the parameters that we used when reporting Tru-cut biopsy specimens of breast cancer patients in routine pathology practice and presented in breast councils may affect lymph node metastasis in T1-2 and clinically/radiologically axilla-negative cases. Using logistic regression analysis, we found that axillary lymph node metastasis was significantly more frequent in patients with tumor size as well as lymphovascular invasion and progesterone receptor expression levels below 80% in multivariate analysis. Although we did not find it significant, we observed axillary lymph node involvement reaching N1-N2-N3 levels, especially in invasive lobular carcinoma tumor histology, in patients with clinically/radiologically reported axilla negative tumor histology. Due to the loss of e-cadherin expression in invasive lobular carcinoma, tumor cells can spread as single cells both within the breast tissue and in the metastatic region. In this respect, as seen in our study, metastasis can be detected in one or more lymph nodes without disrupting the integrity of the parenchyma and without causing a significant change in radiologic appearance. In addition, in our study, we reported more frequent axillary lymph node metastasis in patients with lymphovascular invasion in Tru-cut biopsy specimens. Although tumor cells that invade the vessel are eliminated by immune system cells, the presence of lymphovascular invasion increases the likelihood of lymph node metastasis. A similar association has been reported in other studies [6,25,26]. However, it should be kept in mind that this assessment has limitations when applied to Tru-cut biopsy specimens, as lymphovascular invasion assessment is performed in peritumoral breast parenchyma outside the invasive tumor [27].

Breast cancer includes several molecular subtypes: 70% are ER-positive, 10–15% are Her-2 positive, and 20% are triple-negative [28]. Estrogen and progesterone are steroid hormones found in breast epithelium, regulating mammary gland development via ER/PR. PR synthesis depends on estrogen presence, and estrogen activity is modulated by progesterone, with interactions between them showing synergy or antagonism.

According to SEER data, among 666,852 hormone receptor-positive breast cancer patients, 82.9% are ER-positive/PR-positive, 15.0% are ER-positive/PR-negative, and 2.0% are ER-negative/PR-positive [15]. While ER/PR-positive tumors have a better prognosis, ER-positive, PR-negative cases exhibit poorer cancer-specific survival [14,15,29]. In cases with absent or low PR levels, ER’s functionality may be compromised. Therefore, the efficacy of hormonal therapy can be reduced, as shown in several studies [30,31]. Low or absent PR expression may benefit more from chemotherapy in pre- and perimenopausal ER-positive patients [31].

In our study, we retrospectively examined progesterone receptor expression levels in 199 cases of luminal type, Her-2-negative, clinically and radiologically axilla-negative T1-T2 breast cancer. Progesterone receptor expression levels below 80% have been found to be a factor that increases the risk of lymph node metastasis, along with increasing tumor size and the presence of lymphovascular invasion.

In breast cancer patients, there is no consensus on a threshold value for progesterone receptor to predict the risk of axillary lymph node metastasis. In our study, we analyzed each group of progesterone receptor levels of 10%, 20%, 30%, 40%, 50%, 60%, 70%, and below 80%. While we anticipated that decreased PR levels, especially below 20%, would be associated with an increased risk of lymph node metastasis, we only found significance in the groups with levels below 80%. To highlight that the 10%, 20%, and 50% levels were also examined, we included this information in the study.

The significance of this study is that it provides guidance to both surgeons and pathologists who perform frozen section evaluations with an idea about the likelihood of axillary lymph node metastasis using a parameter routinely assessed from breast Tru-cut biopsy material. Predicting the high likelihood of a positive sentinel lymph node and, therefore, the need for axillary dissection preoperatively will provide convenience to both the surgeon and the pathologist. In our opinion, the importance of the progesterone receptor should be further emphasized due to its ease of assessment in nearly all centers and its cost-effectiveness.

Our study has inherent limitations due to its retrospective nature. This study does not include survival analysis because our aim was to determine whether decreasing progesterone levels have a predictive value for axillary lymph node metastasis. Another limitation could be the relatively small number of patients. The subgroup analysis may demonstrate more significant results with a larger cohort size.

Despite the limitations of this study, working with an experienced pathologist and including a clinically homogeneous group of early-stage patients were key determinants. According to our knowledge, our study presents novel data in terms of evaluating different progesterone levels.

## 5. Conclusions

Our study has determined that the expression level of the progesterone receptor can be used as a parameter to predict the risk of axillary metastasis prior to surgery. In cases of clinically and radiologically axilla-negative, early-stage breast cancer, a progesterone receptor level below 80% in Tru-cut biopsy increases the risk of axillary lymph node metastasis. We believe that by evaluating the progesterone receptor level along with other clinicopathological factors prior to surgery, axillary management during surgery can be better determined.

## Figures and Tables

**Table 1:** Clinical and histological characteristics of the study population, N = 199 patients.

Category	Feature	Value
Patient Demographics	Age (Median)	56 years [35–86 years]
	Age Group	≤40 years: 9 (4.5%) >40 years: 190 (95.5%)
	Menopausal Status	Premenopausal: 80 (40.2%) Postmenopausal: 119 (59.8%)
Tumor Characteristics	Tumor Location	Right-sided: 96 (48.2%) Left-sided: 103 (51.8%)
	Number of Tumors	Unifocal: 185 (93%) Multifocal: 11 (5.5%) Multicentric: 3 (1.5%)
	Histological Type	IDC: 179 (89.9%) ILC: 5 (2.5%) Mixed type: 2 (1%) Others: 13 (6.5%)
	Tumor Grade	Grade I: 25 (12.6%) Grade II: 114 (57.3%) Grade III: 10 (5%) Unknown: 50 (25.1%)
	Lymphovascular Invasion	Present: 59 (29.6%) Absent: 140 (70.4%)
	Perineural Invasion	Present: 56 (28.1%) Absent: 143 (71.9%)
	Extracapsular Invasion	Present: 25 (12.6%) Absent: 174 (87.4%)
	Tumor Size (median)	20 mm (3–50)
	T1 Tumor	101 (50.8%) T1a: 9 (4.6%), T1b: 12 (6%) T1c: 80 (40.2%)
	T2 Tumor	98 (49.2%)
Pathological Nodal Status	Nodal Status	N0: 140 (70.4%) N1: 45 (22.6%) N2: 9 (4.5%) N3: 5 (2.5%)
Surgical Intervention	Type of Surgery	Mastectomy: 16 (8%) Breast-conserving surgery: 183 (92%)
	Axillary Surgery	SLNB: 146 (73.4%) AD: 53 (26.6%) 37 SLNB + complementary AD, 16 due to undetectable SLN
Lymph Node Status	LN Metastasis in SLNB	Positive: 52 (28.4%) Negative: 131 (71.6%)
	LN Metastasis in AD	Positive: 26 (49%) Negative: 27 (51%)
	LN total	Positive: 59 (29.6%) Negative: 140 (70.4%)
Molecular and Hormonal Status	Ki-67 Index	<14%: 75 (37.7%) ≥14%: 112 (56.3%) Unknown: 12 (6%)
	Molecular Subtype	Luminal A: 66 (33.2%) Luminal B: 121 (60.8%) Unknown: 12 (6%)
	Immunohistochemistry	ER+/PR+: 185 (93%) ER+/PR−: 14 (7%)
	Progesterone Receptor	<10%: 21 (10.6%) ≥10%: 178 (89.4%) <20%: 29 (14.6%) ≥20%: 170 (85.4%) <50%: 46 (23.1%) ≥50%: 153 (76.9%) <80%: 71 (35.7%) ≥80%: 128 (64.3%)

IDC: invasive ductal carcinoma; ILC: invasive lobular carcinoma; SNLB: sentinel lymph node biopsy; SLN: sentinel lymph node; AD: axillary dissection; LN: lymph Node; ER/PR: estrogen receptor/progesterone receptor.

**Table 2:** Relationship between risk factors and lymph node status. N = 199 patients. *p* < 0.05 was considered statistically significant.

Variable	Lymph Node-Negative *n* (%)	Lymph Node-Positive *n* (%)	Total N	Chi-Square Value (df)	*p*-Value
Age Group				0.249 (1)	0.618
≤40 years	7 (77.8)	2 (22.2)	9		
>40 years	133 (70.0)	57 (30.0)	190		
Postmenopausal				0.522 (1)	0.470
Yes	86 (72.3)	33 (27.7)	119		
No	54 (67.5)	26 (32.5)	80		
Localization				0.206 (1)	0.650
Right	69 (71.9)	27 (28.1)	96		
Left	71 (68.9)	32 (31.1)	103		
Tumor size group				15.417 (1)	<0.001
≤2 cm	83 (83.0)	17 (17.0)	100		
>2 cm	57 (57.6)	42 (42.4)	99		
Pathological T				19.630 (4)	<0.001
T1a	7 (77.8)	2 (22.2)	9		
T1b	12 (100.0)	0 (0.0)	12		
T1c	65 (81.3)	15 (18.8)	80		
T2	56 (57.7)	41 (42.3)	97		
T3	0 (0.0)	1 (100.0)	1		
Number of Foci				5.624 (2)	0.060
Unifocal	134 (72.4)	51 (27.6)	185		
Multifocal	5 (45.5)	6 (54.5)	11		
Multicentric	1 (33.3)	2 (66.7)	3		
Histological Type				8.8189 (1)	0.42
IDC	126 (70.4)	53 (29.6)	179		
ILC	1 (20)	4 (80)	5		
Others	11 (84.6)	2 (15.4)	13		
Mixed	2 (100)		2		
Lymphovascular Invasion				58.513 (1)	<0.001
Yes	19 (32.2)	40 (67.8)	59		
No	121 (86.4)	19 (13.6)	140		
Grade				2.852	0.415
1	21 (84.0)	4 (16.0)	25		
2	78 (69.0)	35 (31.0)	113		
3	6 (60.0)	4 (40.0)	10		
Unknown	35 (70.0)	15 (30.0)	50		
Ki67 Level				1.270 (1)	0.260
<14%	56 (74.7)	19 (25.3)	75		
≥14%	75 (67.0)	37 (33.0)	112		
PR Level (10)				0.803 (1)	0.370
<10%	13 (61.9)	8 (38.1)	21		
≥10%	127 (71.3)	51 (28.7)	178		
PR Level (20)				0.380 (1)	0.537
<20%	19 (65.5)	10 (34.5)	29		
≥20%	121 (71.2)	49 (28.8)	170		
PR Level (50)				1.532 (1)	0.216
<50%	29 (63)	17 (37)	46		
≥50%	111(72.5)	42 (27.5)	153		
PR Level (80)				5.070 (1)	0.024
<80%	43 (60.6)	28 (39.4)	71		
≥80%	97 (75.8)	31 (24.2)	128		
IHC				0.266 (1)	0.606
ER+PR+	131 (70.8)	54 (29.2)	185		
ER+PR−	9 (64.3)	5 (35.7)	14		
Perineural invasion				15.475 (1)	<0.001
Present	28 (50.0)	28 (50.0)	56		
Absent	112 (78.3)	31 (21.7)	143		

IDC: invasive ductal carcinoma; ILC: invasive lobular carcinoma; PR: progesterone receptor; ER: estrogen receptor; IHC: immunohistochemistry.

**Table 3:** Univariate logistic regression analysis results. N = 199 patients. *p* < 0.05 was considered statistically significant.

Variable	Beta (B)	Std. Error (S.E.)	Wald	df	Sig. (*p*-Value)	Odds Ratio (Exp(B))	95%CI	
							Lower	Upper
Age Group	0.405	0.817	0.246	1	0.620	1.500	0.302	7.443
Postmenopausal Status	−0.227	0.315	0.521	1	0.471	1.254	0.677	2.325
Tumor Size Group	−1.280	0.335	14.605	1	<0.001	3.597	1.865	6.944
Lymphovascular Invasion	−2.596	0.372	48.641	1	<0.001	13.333	6.451	27.777
Ki67 Level	−0.374	0.333	1.264	1	0.261	1.453	0.757	2.703
PR10	0.427	0.479	0.794	1	0.373	1.531	0.599	3.921
PR20	−0.265	0.426	0.379	1	0.538	1.300	0.564	2.994
PR50	−0.438	0.355	1.520	1	0.218	1.550	0.772	3.105
PR80	−0.712	0.319	4.989	1	0.026	2.036	1.090	3.802
Perineural Invasion	−1.285	0.336	14.651	1	<0.001	3.610	1.872	6.993
IHC	−0.298	0.581	0.264	1	0.607	1.347	0.431	4.201

PR: progesterone receptor; IHC: immunohistochemistry.

**Table 4:** Multivariate logistic regression analysis results. N = 199 patients. *p* < 0.05 was considered statistically significant.

Variable	Beta (B)	Std. Error (S.E.)	Wald	df	Sig. (*p*-Value)	Odds Ratio (Exp(B))	95%CI	
							Lower	Upper
Tumor Size Group	−1.173	0.399	8.646	1	0.003	3.236	1.479	7.042
LVI	−2.412	0.405	35.470	1	<0.001	1.111	2.439	5.050
PNI	−0.695	0.417	2.777	1	0.096	2.004	0.884	4.545
PR80	−0.837	0.402	4.344	1	0.037	2.309	1.051	5.076

PR: progesterone receptor; IHC: immunohistochemistry.

## Data Availability

The raw data supporting the conclusions of this article will be made available by the authors without reservation.

## Abbreviations

The following abbreviations are used in this manuscript:

AD	Axillary dissection
ER	Estrogen receptor
IDC	Invasive ductal carcinoma
IHC	Immunohistochemistry
ILC	Invasive lobular carcinoma
IRB	Institutional Review Board
LN	Lymph node
LVI	Lymphovascular invasion
PNI	Perineural invasion
PR	Progesterone receptor
SLN	Sentinel lymph node
SLNB	Sentinel lymph node biopsy
